# Peer Review of “Development of a Digital Platform to Promote Mother and Child Health in Underserved Areas of a Lower-Middle-Income Country: Mixed Methods Formative Study”

**DOI:** 10.2196/60429

**Published:** 2024-07-31

**Authors:** Anonymous

**Keywords:** primary health care, mother and child health, community health worker, slums, digital applications, health communication.

*This is a peer review for “Development of a Digital Platform to Promote Mother and Child Health in Underserved Areas of a Lower-Middle-Income Country: Mixed Methods Formative Study.”*

## Round 1 Review

### General Comments

This paper [1] addresses a principal issue, especially for the developing world where the valuable lives of mothers and children can easily be prevented. However, of course, a big challenge in the proposed solution is the availability of Android devices that are also connected to the internet. This is a limitation, therefore, to be added. Another area that needs to be addressed is related to cultural acceptance and sensitivity to using technology, particularly during the prenatal stage. Also, I noted that the diagrams are not clearly developed and placed in the appropriate places. I am happy with the qualitative part but unfortunately not an authority on quantitative; thus, this part should be vetted by a quantitative expert.

### Specific Comments

#### Major Comments

1. This is an excellent topic requiring continuous literature development.

2. The method used is mixed, whereas I would have preferred the total qualitative inquiry considering the set aims and objectives.

3. The authors need to pay attention to the sociocultural realities of the context; therefore, either address them or acknowledge them as limitations.

#### Minor Comments

4. The diagrams need to be appropriately designed and placed in the paper.
